# Supplementary material for: Evidence supported by Mendelian randomization: impact on inflammatory factors in knee osteoarthritis
Source: Front Med (Lausanne). 2024 May 28;11:1382836. doi: 10.3389/fmed.2024.1382836 (PMC11165061; doi:10.3389/fmed.2024.1382836)
Supplement: Supplementary file 1 [file Data_Sheet_1.ZIP › data/GCST90274771_5e6/771.leaveoneout.pdf]

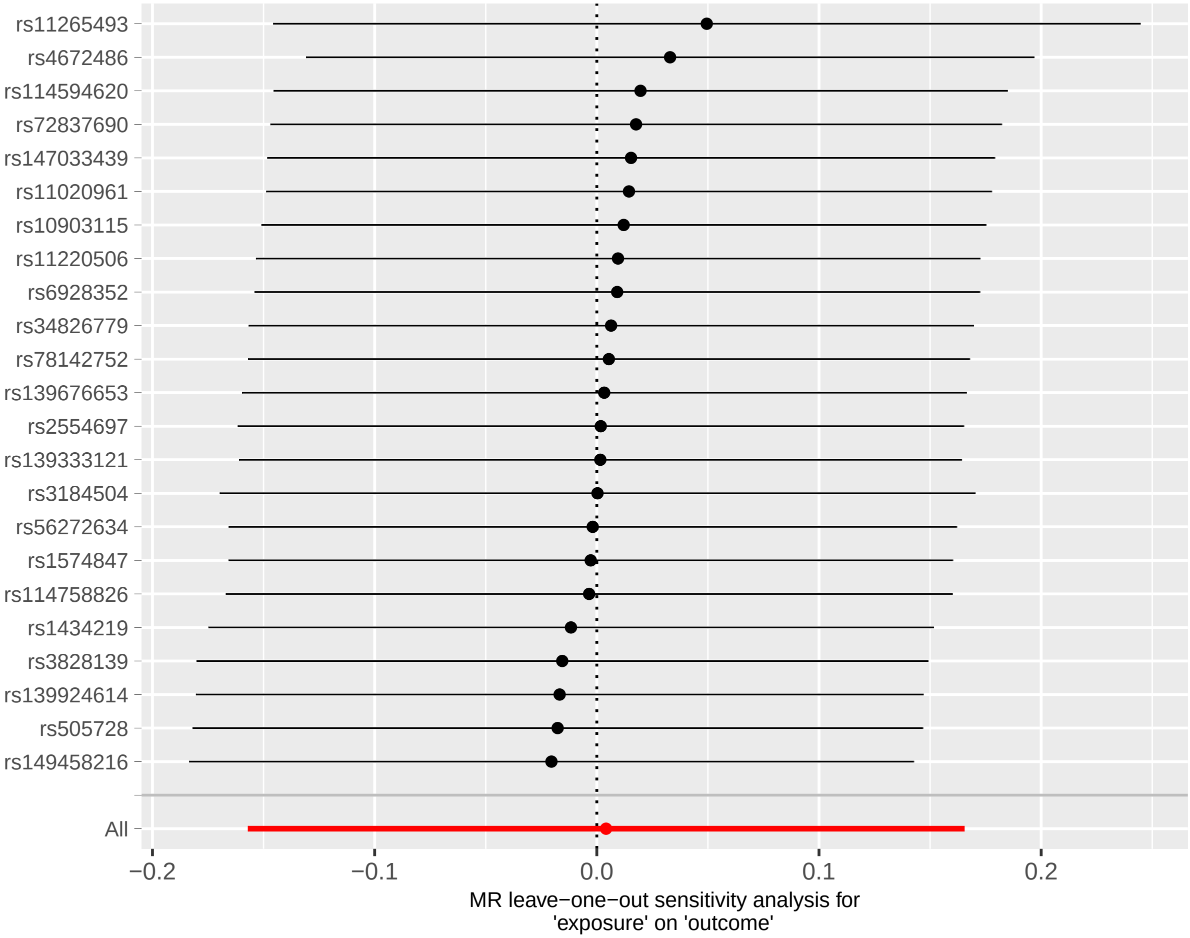
rs11265493
rs4672486
rs114594620
rs72837690
rs147033439
rs11020961
rs10903115
rs11220506
rs6928352
rs34826779
rs78142752
rs139676653
rs2554697
rs139333121
rs3184504
rs56272634
rs1574847
rs114758826
rs1434219
rs3828139
rs139924614
rs505728
rs149458216
All
−0.2
−0.1
0.0
0.1
0.2
MR leave−one−out sensitivity analysis for
'exposure' on 'outcome'
